# How We Made Bread

**Published:** 1875-03

**Authors:** 


### CHAPTER II.

### HOW WE MADE BREAD.

I was indulgent to Alice, and for three days allowed her to wander at will about the grounds and through the maple forest near by. To sit upon the verandas, where clambering roses and sweet honeysuckle scattered perfume on every breeze that stole into the house,—to drink in the song of birds until her heart o'erflowed with music. Then, one morning, interrupting her in a trill, which, for low, sweet melody, rivalled the robbin's song, I said quietly,—

"Alice, my child, come to the kitchen and make the yeast, for to-morrow we bake."

Stifling a half sigh, Alice ran after me, whirling and dancing as she went, and this is the way she made the yeast:

She put her small hand into the bag of hops, and brought it out full of the dried blossoms. These she placed in a tin stew-pan, and pouring upon them a quart of boiling water, left them to boil five minutes, while she stirred to a smooth paste a gill of flour with a little cold water. Then, through a tin strainer, she poured the boiling hop-water slowly upon the paste, stirring continuously that it might grow thin by degrees and perfectly mix with the hop-infusion. She then boiled this thin starch for a few minutes, stirring it while on the range, lest it should settle and burn on the bottom of the pan. Then she returned it to the bowl, adding a large spoonful of salt and the same of white sugar. When this mixture had cooled to blood heat—or, say 98° Fahrenheit—she added a gill of yeast procured from the baker. Stirring all well together, she covered the bowl closely, throwing over it several folds of blanket to protect it from draughts of air, and left it to ferment, or rise. Five or six hours later, finding it getting light, she gave it a good beating, which caused it to settle; and this she repeated several times during the day.

At nine in the evening the yeast was "quiet" enough to be poured into a glass jar covered with an inverted tumbler—in the absence of a glass cap—and set away in the cellar.

Each week during the summer we repeated this process of making yeast, always reserving a gill of the old yeast to start the new, and always washing and scalding the glass jar with care.

It is less trouble to make yeast in this manner, and keep it on hand, than to send to the baker's when it is required; besides, this yeast is superior to that used by most bakers. In winter it will keep two weeks. Glass is better than stone for keeping yeast, being less porous, more easily handled, and more readily cleansed from the old yeast.

At nine o'clock in the evening we set the ferment for the next day's baking. Into a large earthen bowl we put four medium-sized potatoes,—pared, boiled, and mashed, the same as for the table. The water in which they boiled, we drained carefully away. Many cooks use this potato-water to set the ferment, but "the potato, nutritious and harmless as it appears, belongs to a family suspected of very dangerous traits. It is a family connection of the deadly nightshade, and other ill-reputed gentry, and sometimes shows strange proclivities to evil,—now breaking out uproariously, as in the noted potato-rot, and now more covertly in various evil affections. For this reason, scientific directors bid us beware of the water in which potatoes are boiled—into which it appears the evil principle is drawn off—and they caution us not to shred them into stews without previously suffering the slices to lie for an hour or so in salt and water."

To the potato we added a pint of flour, and, gradually, a quart of boiling water, stirring the mixture constantly meanwhile. When all the water was in, the contents of the bowl resembled very thin starch. When the temperature of the ferment had cooled to blood heat, we added a gill of our new yeast, stirred it well, and covered it closely, that it might retain its own warmth unchilled till morning.

Upon entering the kitchen next morning, I found Alice already there.

"Have you looked at the ferment?" I asked. "No, Cousin Kate, I dare not touch anything in your absence, lest I do mischief; but, pray, let me see it. Why, it looks like sea-foam. How very lovely!"

"Yes," I replied, "and were you to taste it, you would find it not unpleasant, but a little sweet and biting to the tongue. And now, from this pan of flour, which has been sifted and warmed, we will add to the ferment a handful at a time, stirring and beating it well meanwhile, until it is thick as batter; then covering, leave it to rise. When light again, and fairly dancing with life (or is it the dance of death?) give it more flour and a good beating. This whipping seems to take all the life out of the batter, but you will find that the dough quickly recovers its old spirits, and is all the lighter for the heavy strokes given it. You will observe, Alice, that I put no salt in the bread, there being enough in the yeast to prevent it from tasting fresh or flat. No one, I think, can like to taste the salt in bread."

"Cousin Kate, why do you set the ferment to rise in an earthern bowl instead of a tin pan or wooden tray?"

"Tin does not protect the ferment from cold air as does this thick earthenware; and wooden vessels are clumsy to handle, and difficult to keep clean and sweet. Bear in mind, Alice, that you can not have perfect success in bread-making, if you allow the ferment or sponge to change temperature, alternating from cold to heat; you must not allow it to take a chill, and then, forcing it into a fever by means of hot-air baths. Such treatment will utterly demoralize any bread-sponge.

"Start your bread at blood-heat, then keep it well covered; and whenever you add flour, let it be of a moderately warm temperature.

"When the sponge is too stiff to be stirred and beaten in this manner, put your hand into it, and work it very thoroughly, adding flour slowly until the dough clings together and seems to have a decided character of its own. The sides of the bowl will then be clean of dough, and you can lift the mass all together, and place it on the bread-board, where you will knead it for half an hour longer, adding flour only as is necessary to keep the dough from sticking to the board or hands.

"When it works clean on the board, and seems lively and spirited, it is well moulded; but test its temper in this way: give the dough a deep poke with your fist, and if it takes the insult meekly and settles down on the board, it is not worked sufficiently; but if, on the contrary, it seems to resent the assault, and springs back after your retreating hand, then it is all right, and you may shape it into loaves.

"Divide the dough into four parts, making each into a round mass; then, by rolling it back and forth under your hand, bring it into a form resembling these bread-pans, which are four or five inches wide, eight or ten long, and six deep; and made, you observe, of sheet-iron, not tin. But for the weight, I would prefer to have them of cast-iron.

"Having warmed and greased the pans, put the loaves in, and set them on the table close together, covering them first with a linen towel; then over that put several folds of woollen blanket or other cloth, reaching to the table on all sides, thus excluding the air from the pans. Bread covered in this way rises evenly, and never has a dried, stiff surface, before it is baked.

"This bread will need to stand in the pans to rise about an hour, perhaps longer, the exact time required depending upon the quantity of flour added at the last kneading, the temperature of the bread when put in the pans, the state of the atmosphere of the room, the size of the loaves, and other circumstances. To decide when the bread is just light enough is a very nice point, and not less important than difficult; for, if you put it to bake a moment too soon, you fail to realize all the good which your labor has entitled you to; while, if you permit it to pass the point of perfect lightness, you lose the best results of your toil.

"Here Alice, read what Miss Beecher and Mrs. Stowe say upon the subject of bread, in their 'American Woman's Home.'"

"The true housewife makes her bread the sovereign of her kitchen—its behests must be attended in all critical points and moments, no matter what else be postponed. She who attends to her bread only when she has done this, and arranged that, and performed the other, very often finds that the forces of nature will not wait for her. The showy mass perfectly mixed, kneaded with care and strength, rises in its beautiful perfection till the moment comes for fixing the air-cells by baking. A few moments now, and the acetous fermentation will begin, and the whole result be spoiled. Many bread-makers pass in utter carelessness over this sacred and mysterious boundary. Their oven has cake in it, or they are skimming jelly, or attending to some other of the so-called higher branches of cookery, while the bread is quickly passing into the acetous stage. At last, when they are ready to attend to it, they find that it has been going its own way; it is so sour that the pungent smell is plainly perceptible. Now, the saleratus-bottle is hauled down and a quantity of the dissolved alkali is mixed with the paste,—an expedient sometimes making itself too manifest by greenish streaks and small acrid spots in the bread. As the result, we have a beautiful article spoiled,—bread without sweetness, if not absolutely sour.

"In the view of many, lightness is the only property required in this article. The delicate, refined sweetness, which exists in carefully kneaded bread, baked just before it passes to the extreme point of fermentation, is something of which they have no conception; and thus they will even regard this process of spoiling the paste by acetous fermentation, and then rectifying that acid by effervescence with an alkali, as something positively meritorious. How else can they relish baker's loaves, such as some produce, drugged with ammonia and other disagreeable things; light, indeed,—so light that they seem to have neither weight nor substance, but with no more sweetness than so much cotton wool?

"Some persons prepare bread for the oven by simply mixing it in the mass, without kneading, pouring it into pans, and suffering it to rise there. The air-cells in bread thus prepared are coarse and uneven; the bread is as inferior in delicacy and nicety to that which is well kneaded, as a raw servant to a perfectly educated and refined lady. The process of kneading seems to impart an evenness to the minute air-cells, a firmness of texture, and a tenderness and pliability to the whole substance, that can be gained in no other way.

"The divine principle of beauty has its reign over bread as well as over all other things; it has its laws of æsthetics; and the bread which is so prepared that it can be formed into separate and well-proportioned loaves, each one carefully worked and moulded, will develop the most beautiful results.

"After being moulded, the loaves should stand usually not over ten minutes,—just long enough to allow the fermentation going on in them to expand each little air-cell to the point at which it stood before it was worked down, and then they should be immediately put into the oven.

"Many a good thing, however, is spoiled in the oven. One thing, however, may be borne in mind as a principle: that the excellence of bread in all its varieties—plain or sweetened—depends on the perfection of its air-cells, whether produced by yeast, egg, or effervescence; that one of the objects of baking is to fix these air-cells; and that the quicker this can be done through the whole mass, the better will the result be. When cake or bread is made heavy by baking too quickly, it is because the immediate formation of the top crust hinders the exhaling of the moisture in the centre, and prevents the air-cells from cooking. The weight, also, of the crust pressing down on the doughy air-cells below, destroys them,—producing that horror of good cooks, a heavy streak. The problem in baking, then, is the quick application of heat, rather below than above the loaf, and its steady continuance till all the air-cells are thoroughly dried into permanent consistency. Every housewife must watch her own oven to know how this can be best accomplished."

"Cousin Kate, Miss Beecher says that after being put in the pans, the bread should stand not more than ten or fifteen minutes to rise, but you say an hour."

"Miss Beecher referred to bread which is allowed to rise in mass, after all the flour is in; and which is kneaded the last time only just enough to shape it into loaves; but in this bread which we are now baking, we dispense with this last moulding, as I think the bread sweeter without it. In the earlier stages of the process there is no danger of working bread too much; but, after all the flour is in, and has been well worked and perfectly fermented, the less it is manipulated and the sooner it is put to bake, the better."

"Cousin Kate, why do you prefer these small pans for baking, to a large one, which would hold all four of the loaves?"

"For the reason given in the extract just read, that 'the excellence of bread depends on the perfection of its air-cells, that one of the objects of baking is to fix these air-cells, and that the quicker this can be done through the whole mass, the better will the result be.'

"Therefore, bake in small loaves, and let the loaf touch the pan at all points,—not another loaf. I have another reason, because it gives more crust; and crust is not only palatable, but a physiological necessity. Remembering that many a good thing is spoiled in the oven, when you put the bread in the pans to rise, look after the range fire."

"Cousin Kate, to return to the bread which is rising, how am I to know when that critical moment of perfect and exact lightness has come? You have warned me against putting it to bake too soon, and cautioned me not to allow it to stand too long; Miss Beecher has also discoursed eloquently upon this fine point; but neither you nor she has explained the signs and signals that shall proclaim, 'behold, this bread is at the supreme moment of its existence; it has risen to its best and highest point of perfection.'"

"That, Alice, is a difficult thing to do, so much depends upon the close observation and fine discrimination of the baker. But when you put bread in the condition of this into the pans, notice the size of the loaf and allow it in rising to double its bulk. When light, if you press your finger gently upon the top of the loaf it will feel soft and spongy beneath. Press the loaf away slightly from the side of the pan in such manner that you can see the texture or grain of the loaf; and, if it looks light, put it into the oven, for I would have it bake while rising toward perfection unattained rather than having fallen from grace. The taste of the first suggests a better state as possible; while the last is hopelessly flat, stale, and unprofitable.

"The bread should be in the oven ten or fifteen minutes before it shows any little brown spots on its surface; and if these appear too soon, lessen the heat,—not by opening the oven-door, but by regulating the damper and draughts of the range. If the bread is not over light when put in the oven, it had better bake too slowly at first than too quickly; and the heat should be created directly under the loaf.

"After the bread is half baked, the heat of the oven should decrease gradually, so that the last of the baking may be done very moderately. Be very careful not to remove your bread from the oven until it is well done, or perfectly "soaked," as the Southern cooks say. When done, the crust should be firm, and equally brown all over. It should be a rich russet-gold, on the verge of a browner tinge,—not that pale sickly hue suggesting a dull fire, and general delapidation. To determine if the bread is done, wet your finger, and touch the bottom of the pan outside; if it hisses, the bread is done.

"My grandmother's test was to touch the bottom of the loaf to the end of her nose; if it burned her nose, the bread was not done.

"You can also judge from the lightness of the loaf,—as well-done bread is much lighter than before.

"Upon removing the bread from the oven, take it out of the pans and tilt it against something upon the table, leaving as much of the surface exposed to the air as possible. Turn it after a few minutes, lest it sweat and soften where it rests. Cover it with nothing so long as it is too hot to be molested by flies; and, as it cools, if necessary, throw over it a very light bread-cloth. When quite cold, put the bread into the bread-box, which should be of tin, or wood lined with zinc,—in a square form, and with a convenient cover. Do not keep bread in the cellar or other damp place, nor in a closet where there is the odor of preserves or pickles. Taken care of in this way, bread will keep perfectly for several days, and I think is better the second or third day after baking than the day it is baked. Never allow the loaves of bread to be cut while warm; for such use, bake in rolls or French twists, and be very careful to have the bread-box emptied of all stale pieces or crumbs, and perfectly clean and dry when the new bread is put away. But this will suffice for our lesson to-day."

